# Epidemiology of heart failure hospitalization in patients with stable atherothrombotic disease: Insights from the TRA 2°P‐TIMI 50 trial

**DOI:** 10.1002/clc.23843

**Published:** 2022-07-19

**Authors:** Benjamin L. Freedman, David D. Berg, Benjamin M. Scirica, Erin A. Bohula, Erica L. Goodrich, Marc S. Sabatine, David A. Morrow, Marc P. Bonaca

**Affiliations:** ^1^ Department of Medicine Beth Israel Deaconess Medical Center, Harvard Medical School Boston Massachusetts USA; ^2^ TIMI Study Group Brigham and Women's Hospital, Harvard Medical School Boston Massachusetts USA; ^3^ CPC Clinical Research University of Colorado School of Medicine Aurora Colorado USA

**Keywords:** diabetes, heart failure, polyvascular disease, risk factors, stable atherosclerosis, TIMI risk score for secondary prevention, vorapaxar

## Abstract

**Background:**

Heart failure (HF) is a growing public health problem and ischemic heart disease is an important risk factor. Understanding the epidemiology of HF in patients with atherosclerosis may help identify subgroups at greater risk who have the potential to derive greater benefit from preventive strategies.

**Methods and Results:**

The TRA 2°P‐TIMI 50 trial randomized 26,449 patients with stable atherosclerosis to the antiplatelet agent vorapaxar versus placebo. Hospitalization for HF (HHF) endpoints were adjudicated from serious adverse events by blinded structured review using established definitions. HHF incidence was estimated using Kaplan–Meier analysis. Independent predictors of HHF risk were identified using multivariable logistic regression. The effect of vorapaxar on HHF risk was explored using Cox regression. The estimated incidence of HHF at 3 years was 1.6%. Independent predictors of HHF included prior HF (adjusted odds ratio [adj‐OR]: 8.31; 95% confidence interval [CI]: 6.56–10.54), age (adj‐OR [per 10 years]: 1.67; 95% CI: 1.47–1.89), type 2 diabetes mellitus (T2DM; adj‐OR: 2.55; 95% CI: 2.01–3.24), polyvascular disease (two‐territory disease, adj‐OR: 1.89; 95% CI: 1.46–2.44; three‐territory disease, adj‐OR: 2.68; 95% CI: 1.94–3.70), chronic kidney disease (CKD; adj‐OR: 1.65; 95% CI: 1.30–2.11), body mass index (BMI; adj‐OR [per 5 kg/m^2^]: 1.15; 95% CI: 1.03–1.27), prior myocardial infarction (MI) (adj‐OR: 1.35; 95% CI: 1.03–1.78), and hypertension (adj‐OR: 1.44; 95% CI: 1.02–2.04). Patients who experienced HHF during follow‐up had higher rates of subsequent rehospitalization and death. Vorapaxar did not modify the risk of HHF.

**Conclusions:**

In patients with stable atherosclerosis, prior HF, age, T2DM, polyvascular disease, CKD, BMI, prior MI, and hypertension are important predictors of HHF risk.

## 1 INTRODUCTION

The prevalence of heart failure (HF) is increasing. More than six million Americans now have HF, and that number is expected to exceed eight million by the year 2030.^1^ Although long‐term prognosis for patients with HF has improved over time due to advances in care, the estimated 5‐year mortality rate following HF hospitalization remains approximately 40%.^2^ Furthermore, HF results in significant morbidity with adverse effects on physical function and quality of life.

In response to this growing public health problem, recent HF guidelines have placed increasing emphasis on HF prevention with a focus on addressing HF risk factors.^3^ Among the multiple risk factors for HF, including hypertension and valvular heart disease, atherosclerotic cardiovascular disease (ASCVD) is the most significant contributor.^4^ For patients with acute myocardial infarction (MI), HF risk is understood to be related in large part to the degree of ischemic myocardial necrosis.^5^ By contrast, the spectrum and key drivers of HF risk among patients with stable coronary artery disease (CAD) and extracoronary atherosclerosis are less well understood.^6^, ^7^, ^8^, ^9^, ^10^

With the emergence of effective therapies for the treatment and prevention of HF, identifying patients at heightened risk for HF is increasingly important. To date, there are few data describing the incidence and predictors of HF as well as the clinical sequelae of HF in patients with stable ASCVD treated with contemporary medical therapy. In addition, while antithrombotic therapy has been shown to reduce the risk of recurrent MI in patients with symptomatic ASCVD, its impact on HF risk is not well described.

We, therefore, evaluated the incidence and predictors of hospitalization for HF (HHF) as well as the potential clinical sequelae of HHF in a cohort of more than 26,000 patients randomized in the TRA 2°P‐TIMI 50 trial.^11^ In addition, we explored the effect of vorapaxar, a protease‐activated receptor‐1 (PAR‐1) antagonist that reduces atherothrombotic events in stable patients with prior MI or peripheral artery disease (PAD),^12^ on the risk of HHF.

## 2 MATERIALS AND METHODS

### 2.1 Study population

The TRA 2°P‐TIMI 50 trial was a multinational, randomized, double‐blind, placebo‐controlled trial of the antiplatelet agent vorapaxar in 26,449 stable patients with established atherosclerosis, defined hierarchically as MI (*n* = 17,779 [67%]), ischemic stroke (*n* = 4,883 [18%]), or symptomatic lower extremity PAD (*n* = 3,787 [14%]). Patients who qualified for the trial on the basis of a history of MI or stroke had events between 2 weeks and 12 months before enrollment, and those with symptomatic PAD had either intermittent claudication with an ankle–brachial index (ABI) < 0.85 or a previous revascularization for limb ischemia. Because ABIs were assessed in all patients, those with no known PAD and no symptoms but an ABI < 0.90 were defined as “asymptomatic PAD.” Patients were randomized to oral vorapaxar sulfate 2.5 mg daily or placebo and were followed for a median of 30 months. The ethics committee at each participating center approved the protocol. Written informed consent was obtained from all patients. Due to heterogeneity with respect to safety in the qualifying cohorts, the US Food and Drug Administration approved vorapaxar in patients with prior MI or PAD, but determined that it should not be used in patients with prior stroke or transient ischemic attack (TIA).^12^

### 2.2 Adjudication of hospitalization for HF

HF or related symptoms were reported in TRA 2°P‐TIMI 50 by local site investigators as serious adverse events (SAEs). Of reported SAEs, 1,102 were identified by a blinded reviewer as candidates for HHF adjudication using verbatim terms (Supporting Information: Figure S1). Selected event terms included those that referenced HF directly (e.g., “congestive heart failure,” “worsening heart failure”) or captured potential signs or symptoms of HF (e.g., “acute pulmonary edema,” “bilateral leg edema,” “dyspnea,” “respiratory failure”). Events identified based on these terms were then adjudicated using the definitions below.

We designed a hierarchical definition of HHF with five mutually exclusive categories. The American Heart Association/American College of Cardiology report on Key Data Elements and Definitions for Cardiovascular Endpoint Events in Clinical Trials^13^ were adapted to define a category of “definite HHF,” and these criteria were broadened to define categories with decreasing specificity: “probable HHF,” “possible HHF,” “HHF not excluded,” and “HHF excluded” (Supporting Information: Methods). SAE reports including event terms, narratives, concomitant medications, and other associated AEs were adjudicated in parallel by two independent reviewers, by applying the hierarchical HHF definition. To maximize clinical information, additional source documents (e.g., those associated with previously adjudicated non‐HHF endpoints) were also reviewed for patients initially categorized as probable HHF, possible HHF, or HHF not excluded. Discrepancies in adjudication results between the two independent reviewers were resolved by group review, including a senior cardiovascular specialist.

### 2.3 Statistical analysis

The primary endpoint for this analysis was defined as the composite of definite or probable HHF (labeled “HHF”). A broader secondary composite endpoint of definite, probable, or possible HHF was also defined. Baseline characteristics of patients who experienced or did not experience HHF during trial follow‐up were compared using Pearson *χ* ^2^ test for categorical variables and Wilcoxon rank‐sum test for continuous variables, as appropriate. HHF incidence was described in the overall cohort and according to potential risk predictors of HHF. HHF incidence was reported using 3‐year Kaplan–Meier (KM) estimates. Univariable risk indicators of HHF were selected from a list of 16 baseline characteristics, and those achieving statistical significance at a threshold of *p* < .05 were included in a multivariable logistic regression model. Independent risk predictors of HHF were then selected using a backward elimination procedure with a significance threshold of *p* < .05.

In addition to assessing the risk of HHF stratified by the strongest independent predictors of HHF risk, we assessed HHF risk in patients: (1) with qualifying MI versus ischemic stroke versus PAD; and (2) with low versus medium versus high atherothrombotic risk based on the TIMI Risk Score for Secondary Prevention (TRS 2°P).^14^ Analyses using TRS 2°P were restricted to the subgroup of patients with a qualifying diagnosis of MI and no prior history of stroke or TIA.

Outcomes after HHF were described based on events occurring between the time of the first HHF event and the end of the study period. Outcomes of interest included admission to an intensive care unit (ICU), in‐hospital death, failure to be discharged home, all‐cause rehospitalization, and postdischarge mortality from a cardiovascular cause or any cause.

Efficacy analyses of vorapaxar were conducted based on intention to treat. Consistent with the primary analysis plan for the trial, HHF incidence was compared between vorapaxar and placebo in all randomized participants using a Cox regression model, with qualifying diagnosis (MI, ischemic stroke, or PAD) and intent to use a thienopyridine as covariates. All analyses were conducted with SAS version 9.4 (SAS Institute).

## 3 RESULTS

### 3.1 Incidence and predictors of hospitalization for HF

Baseline characteristics are summarized in Table 1. Of the 26,449 patients enrolled in the TRA 2°P‐TIMI 50 trial, 2,070 (8%) had a prior history of HF. Baseline left ventricular ejection fraction (LVEF) was documented in 1,755 (85%) of the patients with prior HF. Of these 1,755 patients, the majority (77%) had reduced LVEF (<55%), while the remaining 23% had preserved LVEF (≥55%). A total of 353 patients had at least one HHF event during trial follow‐up (KM event rate = 1.6% at 3 years), and there were 469 total HHF events (inclusive of recurrent events). Age, body mass index (BMI), hypertension, hyperlipidemia, type 2 diabetes mellitus (T2DM), prior HF, PAD, prior MI, prior coronary revascularization, chronic kidney disease, and polyvascular disease were all associated with the risk of HHF (Table 1). The strongest *independent* predictors of HHF risk were prior history of HF, older age, T2DM, and polyvascular disease, defined as having ≥2 symptomatic beds of atherosclerosis (CAD, cerebrovascular disease [CVD], or PAD) (Table 2).

**Table 1:** Baseline characteristics of patients who experienced versus did not experience hospitalization for HF during trial follow‐up.

Characteristic	Hospitalized for HF (*N* = 353)	Not hospitalized for HF (*N* = 26 096)	*p* value
Demographics			
Age (years), median (IQR)	70 (61, 77)	61 (53, 69)	<.001
Female sex (%)	28.0	23.9	.067
White race (%)	85.6	87.3	.31
BMI (kg/m^2^), median (IQR)	28.7 (25.7, 35.2)	27.6 (24.9, 30.8)	<.001
Clinical characteristics			
Current smoker (%)	17.6	20.8	.13
Hypertension (%)	87.8	68.5	<.001
Hyperlipidemia (%)	90.7	83.1	<.001
Diabetes mellitus (%)	62.0	24.9	<.001
Prior HF (%)	55.2	7.2	<.001
Prior stroke or TIA (%)	23.5	23.7	.93
Prior CAD (%)	91.8	78.1	<.001
Prior MI (%)	77.9	72.4	.021
Prior PAD (%)	51.8	21.7	<.001
Prior coronary revascularization (%)	74.5	65.2	<.001
eGFR < 60 ml/min/1.73 m^2^ (%)	46.6	15.2	<.001
Atherosclerosis affecting			
One vascular bed (%)	41.1	77.1	<.001
Two vascular beds (%)	38.8	18.5	<.001
Three vascular beds (%)	20.1	4.5	<.001
Baseline medical therapy			
Aspirin (%)	91.8	93.5	.18
Statin (%)	88.7	89.8	.50
ACEI or ARB (%)	78.2	74.0	.074

*Note*: Baseline characteristics of patients who experienced or did not experience HHF during trial follow‐up were compared using Pearson *χ* ^2^ test for categorical variables and Wilcoxon rank‐sum test for continuous variables, as appropriate.

Abbreviations: ACE, angiotensin‐converting enzyme; ARB, angiotensin receptor blocker; BMI, body mass index; CAD, coronary artery disease; eGFR, estimated glomerular filtration rate; HF, heart failure; HHF, hospitalization for heart failure; IQR, interquartile range; MI, myocardial infarction; PAD, peripheral artery disease.

**Table 2:** Independent predictors of risk for HHF in patients with stable atherosclerosis.

Characteristic	*χ* ^2^	Adjusted OR (95% CI)
History of HF	306.2	8.31 (6.56–10.54)
Age (10‐year increase)	62.2	1.67 (1.47–1.89)
Type 2 diabetes mellitus	58.8	2.55 (2.01–3.24)
Polyvascular disease with three vascular beds (vs. one)	36.0	2.68 (1.94–3.70)
Polyvascular disease with two vascular beds (vs. one)	23.7	1.89 (1.46–2.44)
eGFR < 60 ml/min/1.73 m^2^	16.3	1.65 (1.30–2.11)
BMI (5 kg/m^2^ increase)	6.3	1.15 (1.03–1.27)
Prior myocardial infarction	4.5	1.35 (1.03–1.78)
History of hypertension	4.4	1.44 (1.02–2.04)

Abbreviations: BMI, body mass index; CI, confidence interval; eGFR, estimated glomerular filtration rate; HF, heart failure; HHF, hospitalization for heart failure; OR, odds ratio.

**Figure 1:**
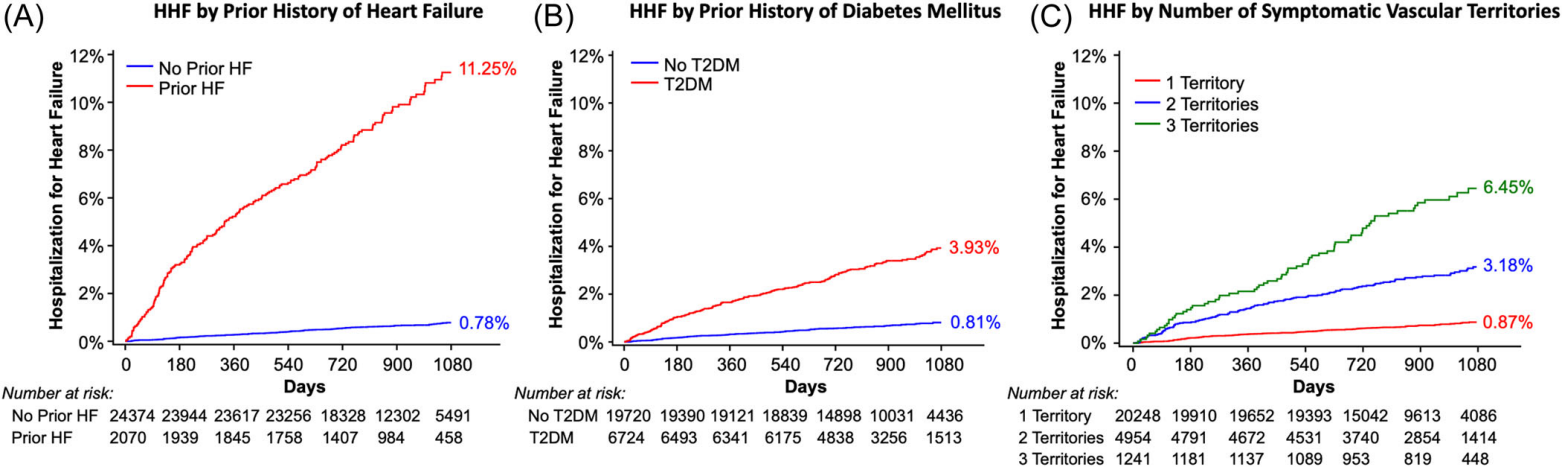
Cumulative incidence of hospitalization for heart failure: (A) in patients with versus without prior heart failure; (B) in patients with versus without T2DM; and (C) by the extent of atherosclerosis. Cumulative incidence rates are expressed using Kaplan–Meier estimates. Vascular territories are defined here as the coronary, peripheral, and cerebral arterial systems. HF, heart failure; HHF, hospitalization for heart failure; T2DM, type 2 diabetes mellitus.

After multivariable adjustment, patients with a prior history of HF had a >8‐fold higher rate of HHF compared to those with no prior history of HF (KM rate 11.2% vs. 0.8% at 3 years; *p* < .01) (Figure 1A), and patients with T2DM had a >2.5‐fold higher rate of HHF than those without T2DM (3.9% vs. 0.8% at 3 years; *p* < .01) (Figure 1B). HHF risk was also highly associated with the burden of symptomatic atherosclerosis—patients with three symptomatic vascular territories had an HHF event rate of 6.5% at 3 years, as compared with 3.2% in those with two symptomatic territories, and 0.9% in those with one symptomatic territory (*p*‐trend < 0.01; Figure 1C).

**Figure 2:**
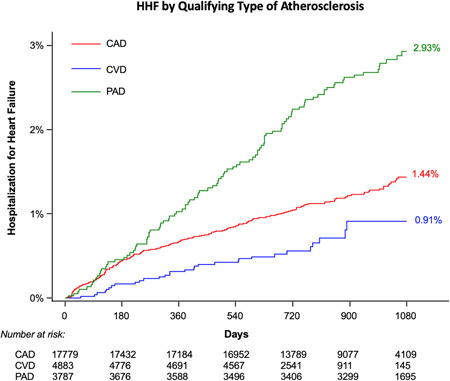
Cumulative incidence of new or recurrent hospitalization for heart failure by qualifying type of atherosclerosis. Cumulative incidence rates are expressed using Kaplan–Meier estimates. CAD, coronary artery disease; CVD, cerebrovascular disease; HHF, hospitalization for heart failure; PAD, peripheral artery disease.

The incidence of HHF differed by qualifying disease, with the highest rate seen in those qualifying with PAD (2.9%), followed by those qualifying with MI (1.4%), and then those qualifying with ischemic stroke (0.9%; Figure 2). Among patients with prior MI and no history of stroke or TIA, HHF incidence was higher in patients with higher TRS 2°P scores: patients with ≥3 atherothrombotic risk indicators (high risk) experienced HHF at a rate of 5.4%, compared to 0.5% in those with one to two risk indicators (intermediate risk), and 0.1% in those with no risk indicators (low risk) (*p*‐trend < 0.01; Supporting Information: Figure S2). This trend remained significant when patients with pre‐existing HF were excluded from the analysis (Supporting Information: Figure S3).

### 3.2 Outcomes after hospitalization for HF

**Figure 3:**
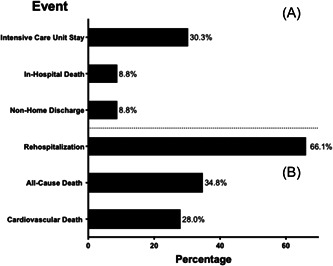
Clinical sequelae among patients experiencing new or recurrent hospitalization for heart failure. (A) In‐hospital outcomes of all patients experiencing an HHF event during follow‐up (*n* = 353). Non‐home discharge denotes patients who were discharged to a rehabilitation center, long‐term care facility, or another acute care hospital. (B) Postdischarge outcomes of patients surviving to hospital discharge (*n* = 322). HHF, hospitalization for heart failure.

Of the 353 patients who experienced an HF hospitalization, the median hospital length of stay was 6 days (interquartile range [IQR]: 4–9 days); 30% required admission to an intensive care unit and 8.8% died before leaving the hospital (Figure 3A). Among patients who survived their initial HF hospitalization (*n* = 322), the rate of rehospitalization for HF was 25% and of rehospitalization for any cause was 66%, with a median time to rehospitalization of 82 days (IQR: 23–232 days). By the end of follow‐up, cardiovascular and all‐cause mortality among patients who experienced an HHF event were 28% and 35%, respectively (Figure 3B). The median time between HHF and death from any cause was 120 days (IQR: 13–413 days).

### 3.3 Vorapaxar and hospitalization for HF

HHF occurred in 189 patients (3‐year KM rate = 1.7%) in the vorapaxar group and 164 patients (3‐year KM rate = 1.5%) in the placebo group (hazard ratio [HR]: 1.15; 95% CI: 0.93–1.42; *p* = .19). No significant differences were seen between the vorapaxar and placebo groups with respect to the incidence of the other HHF endpoints examined (Supporting Information: Table S1). Furthermore, the incidence of HHF did not differ significantly by treatment group within any major subgroup examined (Supporting Information: Figure S4).

## 4 DISCUSSION

This analysis of HHF risk and outcomes in over 26,000 patients randomized in a contemporary clinical trial of secondary prevention with antiplatelet therapy provides several important observations. First, the risk of HHF in patients with stable ASCVD is highly heterogenous with a >8‐fold higher risk in those with a history of HF versus those with no prior HF, a >2.5‐fold higher risk in those with T2DM versus those without, and an approximately 2‐fold higher risk in patients with PAD or polyvascular disease, as compared to those with CAD or ischemic stroke alone. Second, even with current patterns of aggressive risk factor modification and secondary prevention, outcomes in those with atherosclerosis who experience an HF hospitalization are poor, with approximately 10% dying during the index HF hospitalization and approximately one‐third dying by the end of trial follow‐up. Finally, more intensive antiplatelet therapy with vorapaxar, while clearly efficacious for reducing recurrent ischemic events, did not reduce the risk of HHF over a median follow‐up of 30 months.

### 4.1 Risk of hospitalization for HF in patients with stable atherosclerosis

The observed rate of HHF in this study (~0.5%/year) was consistent with other contemporary clinical trial cohorts of patients with stable ASCVD and nearly identical to the adjudicated HHF event rate observed in the FOURIER trial.^15^ This observation suggests that, in this clinical setting, a rigorous structured approach using safety data to ascertain HHF outcomes may yield similar results to conventional prospective event adjudication by a clinical events committee.

Although the overall HHF event rate was relatively low in this selected clinical trial population, our data underscore the heterogeneity in HHF risk among patients with stable atherosclerosis. These findings build on recently developed clinical risk models designed to stratify HHF risk in patients with T2DM and on our previous work using circulating biomarkers to stratify HHF risk in this cohort of patients with stable atherosclerosis.^16^, ^17^ Collectively, these data have important implications for defining populations to be studied and/or treated with HF preventive therapies. For example, sodium‐glucose cotransporter‐2 (SGLT2) inhibitors have recently emerged as an option for reducing the risk of HHF in patients with T2DM and in patients with HF with reduced ejection fraction irrespective of T2DM status.^18^, ^19^, ^20^, ^21^ Our observation that patients with T2DM and patients with prior HF were at particularly high‐risk for HHF in this cohort support the large potential impact of using SGLT2 inhibitors in the populations for which they are currently approved. In addition, our results highlight that polyvascular disease is an important independent indicator of HHF risk, raising the hypothesis that SGLT2 inhibitors may have therapeutic potential in such patients, irrespective of their T2DM or prior HF status. This hypothesis deserves further investigation in prospective clinical trials.

### 4.2 Outcomes following hospitalization for HF in patients with stable atherosclerosis

Outcomes in those who experienced HHF were poor. Almost 30% required ICU admission and nearly 10% died during their HF hospitalization. During the remainder of follow‐up (median 386 [IQR: 123–657] days), 66% had a recurrent hospitalization and 35% died following the initial HHF event. These findings indicate that in spite of advances in medical therapy, preventing HHF and its complications remains a significant unmet need.

### 4.3 Effect of antiplatelet therapy on risk of hospitalization for HF

Finally, in this randomized assessment, more intensive antiplatelet therapy with vorapaxar versus placebo did not reduce the risk of HHF overall or in high‐risk subgroups. Given that ischemic heart disease is a leading cause of HF, we hypothesized that the 17% relative reduction in MI risk among vorapaxar‐treated trial participants would translate to a lower risk of subsequent HHF.^11^, ^22^ Moreover, heightened platelet activity leading to chronic coronary microthrombosis has been implicated as a mechanism of HF progression.^23^ The observation that vorapaxar did not reduce HHF risk suggests either that follow‐up duration was insufficient or that the majority of HHF events were precipitated by events other than intercurrent MI.

### 4.4 Limitations

There are several limitations to the current study. Because of the retrospective design, our HHF adjudication relied upon SAE narrative reports with varying degrees of detail; this constraint may have diminished the sensitivity of our HHF event ascertainment. Despite this limitation, the observed rate of HHF in our study was highly comparable to those seen in similar clinical trial populations with prospective adjudication of HHF endpoints. Furthermore, while reduced sensitivity may result in underestimation of HHF event rates, it would not be expected to substantially modify the risk relationships between clinical predictors of HHF risk or the effect of the randomized therapy, vorapaxar, on HHF. In addition, the observations regarding outcomes after HHF do not indicate that HF hospitalization is necessarily the cause of those outcomes, as patients who were hospitalized for HF were clearly sicker than those who were not. Finally, as noted above, although vorapaxar did not decrease HHF risk in this clinical trial cohort, the current study was not powered to detect a difference and thus the current study does not exclude a possible benefit, particularly in higher‐risk patients followed for a longer period of time.

## 5 CONCLUSIONS

Patients with stable atherosclerosis vary in their risk of HHF, with much higher event rates observed in patients with prior HF, T2DM, and polyvascular disease. Outcomes after HF hospitalization are poor, with high rates of recurrent hospitalization and mortality. Prevention of HHF in patients with ASCVD, therefore, appears to be an important therapeutic goal. Despite reducing MI risk, pharmacologic inhibition of PAR‐1 does not appear to have a significant effect on HHF risk over a 3‐year time horizon.

## CONFLICTS OF INTEREST

David D. Berg, Benjamin M. Scirica, Erica L. Goodrich, Erin A. Bohula, Marc S. Sabatine, and David A. Morrow are members of the TIMI Study Group, which has received institutional research grant support through Brigham and Women's Hospital from Abbott, Amgen, Aralez, AstraZeneca, Bayer HealthCare Pharmaceuticals, Inc., BRAHMS, Daiichi Sankyo, Eisai, GlaxoSmithKline, Intarcia, Janssen, MedImmune, Merck, Novartis, Pfizer, Poxel, Quark Pharmaceuticals, Roche, Takeda, The Medicines Company, and Zora Biosciences. David D. Berg has received consulting fees from AstraZeneca. Benjamin L. Freedman has no disclosures. Benjamin M. Scirica has received consultant fees/honoraria from AbbVie, Allergan, Covance, Eisai, Elsevier Practice Update Cardiology, Esperion, Lexicon, Medtronic, Novo Nordisk, Sanofi, AstraZeneca Pharmaceuticals, Biogen Idec, Boehringer Ingelheim Pharmaceuticals, Inc., Dr. Reddy's Laboratories Inc., Forest Laboratories, GE Healthcare, GlaxoSmithKline, Health@Scale, Lexicon, Merck & Co., Inc., and St. Jude Medical; has received institutional research grants from AstraZeneca, Daiichi‐Sankyo, Eisai, Merck, Pfizer, and Poxel; and holds equity in Health@Scale. Erin A. Bohula has received consulting fees from Kowa, Servier, Novo Nordisk, Amgen, PriMed, and Medscape. Marc S. Sabatine has received consulting fees from Alnylam, Anthos Therapeutics, Amgen, AstraZeneca, Bristol‐Myers Squibb, CVS Caremark, Dynamix, Esperion, IFM Therapeutics, Intarcia, Ionis, Janssen Research and Development, The Medicines Company, MedImmune, Merck Sharp & Dohme, and Novartis. David A. Morrow has received grants and personal fees from Abbott Laboratories, AstraZeneca, Roche Diagnostics, and Bayer Pharma; grants from Novartis, Daiichi Sankyo, Eisai, GlaxoSmithKline, Takeda, Pfizer, Quark, The Medicines Company, Merck, and Zora Diagnostics; personal fees from InCarda. Marc P. Bonaca reports receiving grant support from Amgen, AstraZeneca, Merck, Novo Nordisk, Pfizer, and Sanofi.

## Supporting information

Supporting information.

## Data Availability

The data that support the findings of this study are available on reasonable request to the TIMI Study Group. The data are not publicly available due to privacy, contractual, and/or ethical restrictions.
