# Supplementary material for: Epidemiology of heart failure hospitalization in patients with stable atherothrombotic disease: Insights from the TRA 2°P‐TIMI 50 trial
Source: Clin Cardiol. 2022 Jul 19;45(8):831–8. doi: 10.1002/clc.23843 (PMC9346972; doi:10.1002/clc.23843)
Supplement: Supplementary file 1 — Supporting information. [file CLC-45-831-s001.docx]

**SUPPORTING INFORMATION**

**Epidemiology of Heart Failure Hospitalization in Patients with Stable Atherothrombotic Disease: Insights from the TRA 2°P-TIMI 50 Trial**

Short Title: Heart Failure Epidemiology in Stable Atherosclerosis

Authors: Benjamin L. Freedman, MD,^1#^ David D. Berg, MD, MPH,^2#^* Benjamin M. Scirica, MD, MPH,^2^ Erin A. Bohula, MD, DPhil,^2^ Erica L. Goodrich, MS,^2^ Marc S. Sabatine, MD, MPH,^2^ David A. Morrow, MD, MPH,^2^ Marc P. Bonaca, MD, MPH^3^*

^#^*Authors contributed equally to this work*

^1^Department of Medicine, Beth Israel Deaconess Medical Center, Harvard Medical School, 330 Brookline Ave, Boston, MA 02115

^2^TIMI Study Group, Brigham and Women’s Hospital, Harvard Medical School, 60 Fenwood Road, Suite 7022, Boston, MA 02115

^3^CPC Clinical Research, University of Colorado School of Medicine, 13199 E. Montview Blvd, Suite 200, Aurora, CO 80045

*Contact Information for Corresponding Authors:

Marc P. Bonaca, M.D., M.P.H.

Address: CPC Clinical Research

University of Colorado School of Medicine

13199 E. Montview Blvd, Suite 200

Aurora, CO 80045

Email: [Marc.Bonaca@cpcmed.org](mailto:Marc.Bonaca@cpcmed.org)

Telephone: (303) 991-7406

David D. Berg, M.D., M.P.H.

Address: TIMI Study Group

60 Fenwood Road, Suite 7022

Boston, MA 02115

Email: [dberg1@bwh.harvard.edu](mailto:dberg1@bwh.harvard.edu)

Telephone: (617) 278-0145

Table OF CONTENTS

[Supplemental Methods 3](#_Toc80170495)

[Supplementary Table S1 6](#_Toc80170496)

[Supplemental Table S2 7](#_Toc80170497)

[Supplemental Figure S1 8](#_Toc80170498)

[Supplemental Figure S2 9](#_Toc80170499)

[Supplemental Figure S3 10](#_Toc80170500)

[SUPPLEMENTAL FIGURE S4 11](#FigureS4)

Supplemental Methods**: Hospitalization for Heart Failure Endpoint (HHF) Definitions**

*Definite HHF*: meets all listed criteria

1. Hospitalization ≥24 hours (or change in date if admission/discharge times not documented) ***or*** urgent/unscheduled office or emergency department visit
2. Diagnosis of HF by site investigator
3. Has ≥1 of the following HF symptoms:
   1. **Dyspnea** – dyspnea with exertion, dyspnea at rest, orthopnea, paroxysmal nocturnal dyspnea, nocturnal cough in supine position, tachypnea
   2. **Fatigue**
   3. **Reduced exercise tolerance**
   4. **Symptoms relating to decreased end-organ perfusion** - worsening cerebral, renal, liver, abdominal or gastrointestinal, peripheral circulatory function manifested by symptoms such as dizziness, lightheadedness, syncope, confusion, altered mental status, restlessness, decline in cognitive state, nausea, vomiting, abdominal pain, abdominal fullness, abdominal discomfort or abdominal tenderness, cold clammy extremities, discoloration of extremities or lips, jaundice, pain in extremities, reduced urine output, darkening of urine color, chest pain, and/or palpitations
   5. **Other symptoms of volume overload** - swelling of lower extremities; swelling or indentation of pressure marks in areas of fluid accumulation such as the legs, ankles, or lower back; an increase in abdominal girth, right-sided abdominal fullness, discomfort, or tenderness; an increase in body weight; oozing and development of skin breakdown in lower extremities.
4. Has ≥2 of the following HF physical exam findings:
   1. **Elevated jugular venous pressure and/or positive hepatojugular reflux**
   2. **Lung auscultation suggesting pulmonary edema** - Crackles, rales, crepitations, or narrative states that pulmonary edema was found on physical exam
   3. **Peripheral edema** – double-count as “other symptom of volume overload”, and vice versa
   4. **Abdominal distention or ascites** – double-count as “other symptom of volume overload”, and vice versa
   5. **S3 gallop**
   6. **Weight gain** – double-count as “other symptom of volume overload”, and vice versa
   7. **Report of pulmonary edema with no radiographic or auscultatory evidence cited** – assumed to be pulmonary crackles

***or***

Has ≥1 HF physical exam finding and ≥1 of the following non-physical exam objective findings of HF:

1. **Radiographic evidence of pulmonary edema** – chest radiograph or other imaging modality such as computed tomography or magnetic resonance imaging with evidence of pulmonary venous or alveolar congestion, interstitial or pulmonary edema, bilateral pleural effusion, or cephalization of venous flow
2. **Elevated B-type natriuretic peptide (BNP)** - Serum BNP ≥ 100 pg/mL or NT-proBNP ≥ 300 pg/mL
3. **Invasive evidence of HF** - Right heart catheterization demonstrating elevated cardiac filling pressures and/or reduced cardiac index
4. Died within 48 hours of admission (*not* on comfort measures only) ***or*** received one of the following HF treatments:
   1. **Intravenous diuretic**
   2. **Intensified oral diuretic therapy** – increase in oral diuretic dose or addition of another oral diuretic (may be counted as HF treatment only if patient was hospitalized). For this analysis, diuretic therapy of unspecified route of administration was counted as “intensified oral diuretic therapy.”
   3. **Intravenous vasoactive therapy** – inotrope, vasodilator, or vasopressor
   4. **Mechanical fluid removal** – ultrafiltration, hemofiltration, dialysis
   5. **Mechanical circulatory support** – intra-aortic balloon pump, ventricular assist device, extracorporeal membrane oxygenation, total artificial heart

*Probable HHF*: meets all listed criteria

1. Hospitalization ≥24 hours (or change in date if admission/discharge times not documented) ***or*** urgent/unscheduled office or emergency department visit
2. Diagnosis of HF by site investigator
3. Has ≥1 physical exam ***or*** non-physical exam objective finding of HF (see *Definite HHF* criteria)
4. Died within 48 hours of admission (*not* on comfort measures only) ***or*** received HF treatment (see *Definite HHF* criteria), where digoxin also counts as treatment
5. Does not meet criteria for *Definite HHF*

*Possible HHF*: meets all listed criteria

1. Hospitalization ≥24 hours (or change in date if admission/discharge times not documented) ***or*** urgent/unscheduled office or emergency department visit
2. Diagnosis of HF by site investigator ***or*** has ≥1 HF symptom (see *Definite HHF* criteria) ***or*** has HF symptom(s) not otherwise specified
3. Died within 48 hours of admission (*not* on comfort measures only) ***or*** received HF treatment, where digoxin also counts as treatment ***or*** has ≥1 HF physical exam finding ***or*** has ≥1 non-physical exam objective finding of HF (see *Definite HHF* criteria)
4. Does not meet criteria for *Definite HHF*, *Probable HHF*, or *HHF excluded*

*HHF not excluded*: meets all listed criteria

1. Hospitalization ≥24 hours (or change in date if admission/discharge times not documented) ***or*** urgent/unscheduled office or emergency department visit
2. Diagnosis of HF by site investigator ***or*** has ≥1 HF symptom (see *Definite HHF* criteria) ***or*** has HF symptom(s) not otherwise specified
3. Does not meet criteria for *Definite HHF*, *Probable HHF*, *Possible HHF*, or *HHF excluded*

*HHF excluded*: meets ≥1 of the listed criteria

1. Site investigator made no HF diagnosis ***and*** made an alternative diagnosis
2. No HF diagnosis or symptoms (see *Definite HHF* criteria) are reported (including HF symptom[s] not otherwise specified)
3. Admitted for elective placement of implantable cardioverter-defibrillator but without other HF treatment, physical exam findings, non-physical exam objective findings, or new/worsening symptoms (see *Definite HHF* criteria)

Supplementary Table S1**. Effect of Vorapaxar on Incidence of Hospitalization for Heart Failure.**

| End Point | **Vorapaxar (N=13,225)** | **Placebo (N=13,224)** | **Hazard Ratio**  **(95% CI)** | **P Value** |
| --- | --- | --- | --- | --- |
|  | *n (3-year KM rate)* | |  |  |
| Definite or probable HHF | 189 (1.7) | 164 (1.5) | 1.15 (0.93-1.42) | 0.19 |
| Definite, probable, or possible HHF | 281 (2.5) | 255 (2.4) | 1.10 (0.93-1.30) | 0.27 |
| Definite HHF | 96 (0.9) | 78 (0.7) | 1.23 (0.91-1.65) | 0.18 |
| Probable HHF | 123 (1.1) | 104 (1.0) | 1.18 (0.91-1.53) | 0.21 |
| Possible HHF | 109 (1.0) | 120 (1.1) | 0.90 (0.70-1.17) | 0.44 |

HHF = hospitalization for heart failure.

Supplemental Table S2**. Hospitalization for Heart Failure Adjudication Output & Consensus.**

| **HHF Category** | **Adjudicator #1** | **Adjudicator #2** | **Group Consensus** | **Agreement Rate (%)** | **κ** |
| --- | --- | --- | --- | --- | --- |
| Definite | 201 | 171 | 214 | 94 | 0.78 |
| Probable | 260 | 272 | 257 | 90 | 0.74 |
| Possible | 276 | 303 | 260 | 86 | 0.64 |
| Not Excluded | 110 | 138 | 111 | 92 | 0.61 |
| Excluded | 256 | 219 | 260 | 90 | 0.70 |
| Definite or Probable | 461 | 443 | 471 | 92 | 0.84 |
| Definite, Probable or Possible | 737 | 746 | 731 | 91 | 0.79 |
| Total SAEs Reviewed | 1103 | 1103 | 1102^a^ | — | — |

^a^ One SAE was recognized as a duplicate and censored upon group review. HHF = hospitalization for heart failure; SAE = serious adverse event.

Supplemental Figure S1. **Retrospective Adjudication Procedure.**


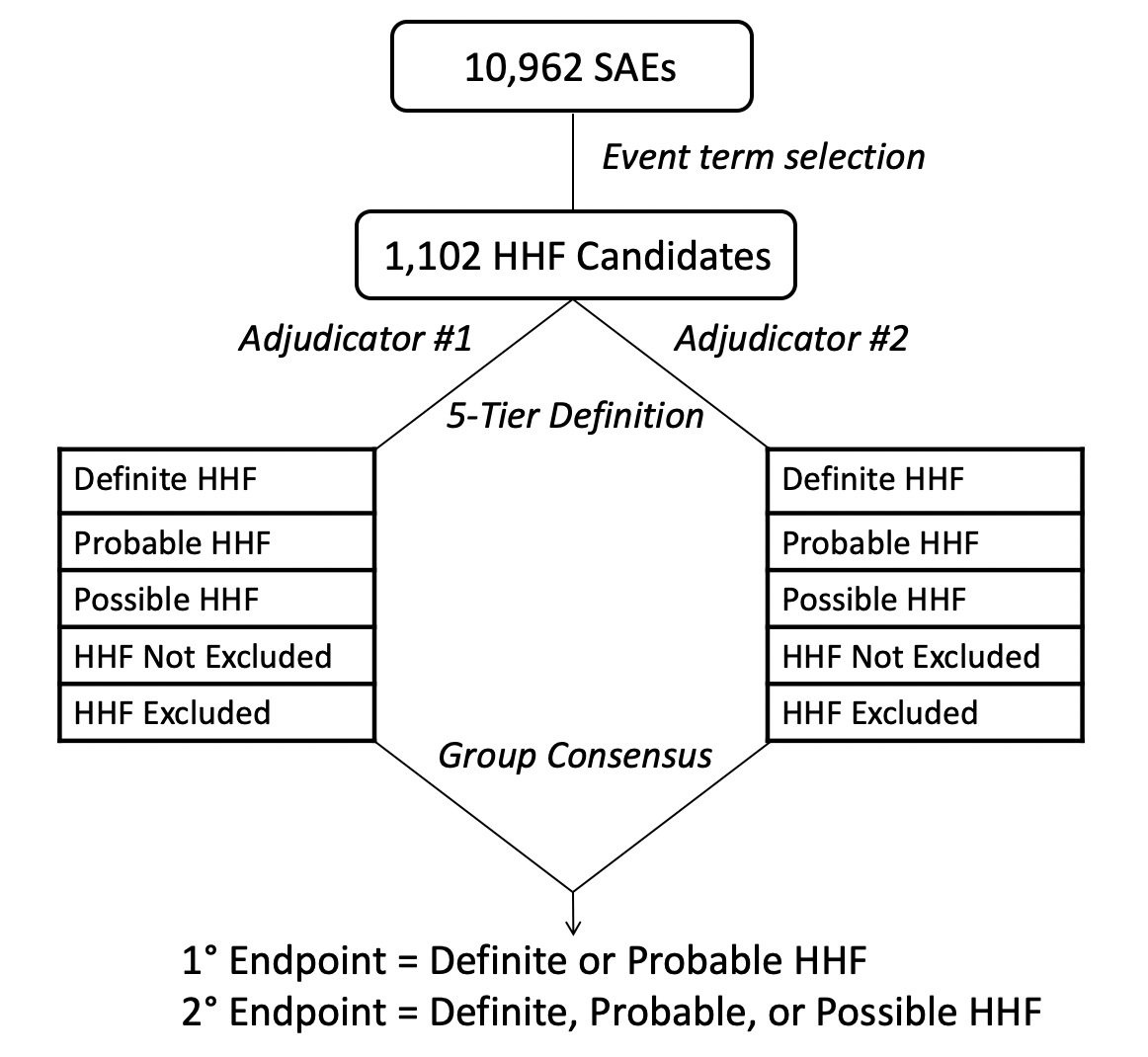


Of the 10,962 serious adverse event verbatim terms with plausible relation to heart failure, 1,102 were selected for adjudication by two independent reviewers using a multi-tiered definition of hospitalization for heart failure. Any discrepancies in HHF adjudication were resolved by consensus. HHF = hospitalization for heart failure.

Supplemental Figure S2**. Incidence of Hospitalization for Heart Failure by TIMI Risk Score for Secondary Prevention (TRS 2°P) risk group.**


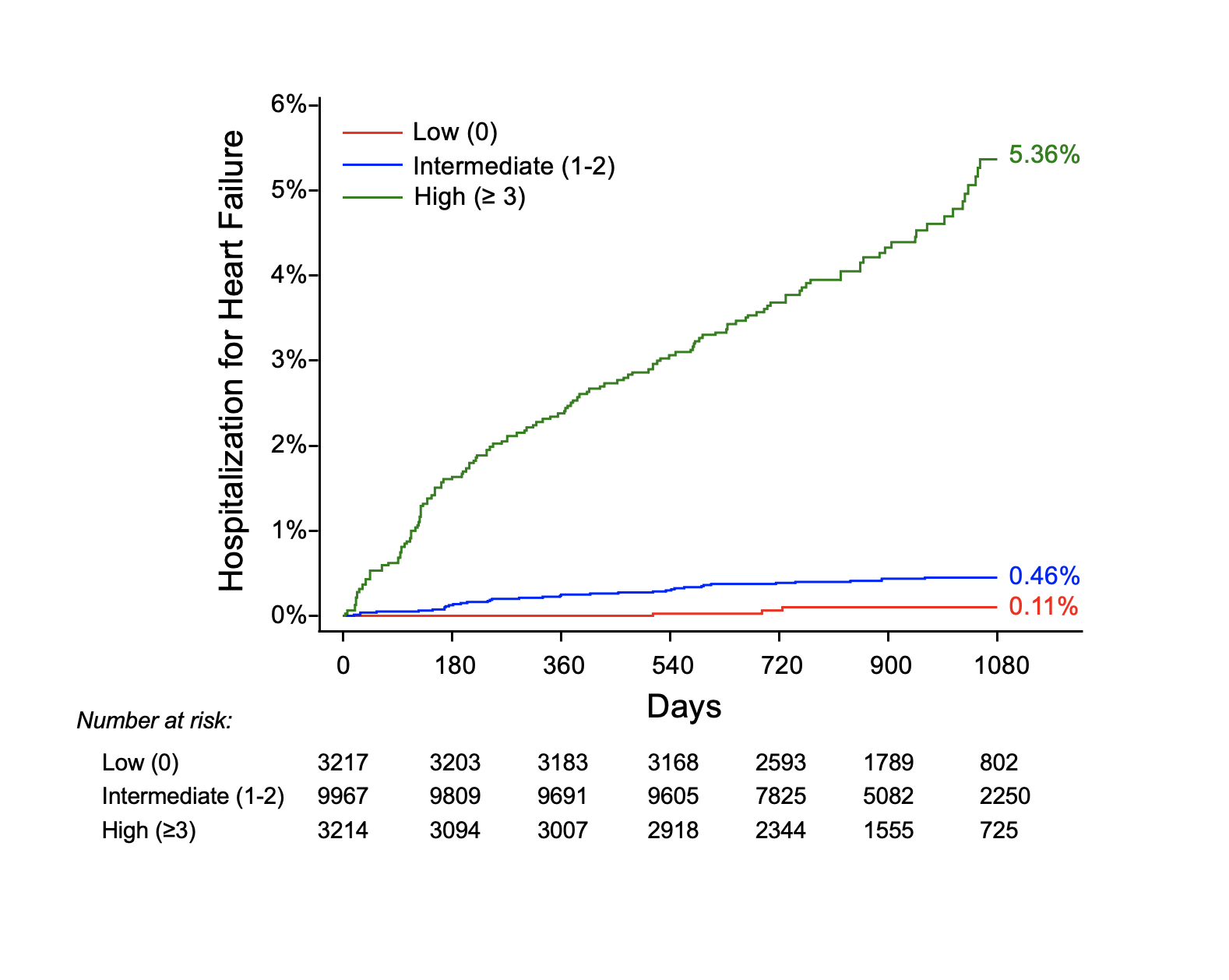


This analysis was restricted to patients qualifying with prior MI. Cumulative incidence rates are expressed using Kaplan-Meier estimates. Low-, intermediate-, and high-risk TRS 2°P risk groups correspond to patients with 0, 1-2, or ≥3 risk factors for recurrent atherothrombosis. These risk factors include heart failure, hypertension, age ≥75 years, diabetes mellitus, prior stroke, prior coronary artery bypass graft, peripheral artery disease, current smoking, and renal dysfunction (estimated glomerular filtration rate <60 ml/min/1.73 m^2^). TRS 2°P = TIMI Risk Score for Secondary Prevention.

Supplemental Figure S3**. Incidence of Hospitalization for Heart Failure by TIMI Risk Score for Secondary Prevention (TRS 2°P) risk group, including in the subgroup with no prior HF.**


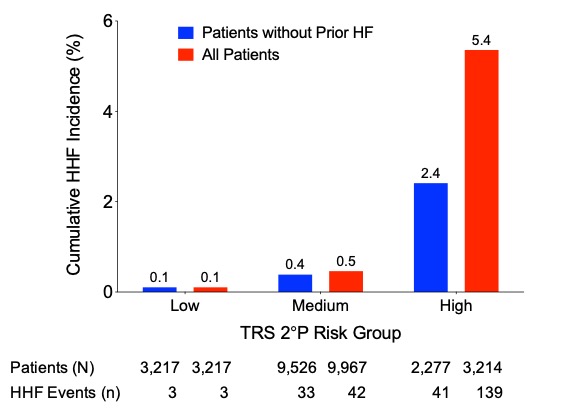


Cumulative incidence rates are expressed using 3-year Kaplan-Meier estimates. Low, intermediate, and high-risk TRS 2°P risk groups correspond to patients with 0, 1-2, or ≥3 risk factors for recurrent atherothrombosis. These risk factors include HF, hypertension, age ≥75 years, diabetes mellitus, prior stroke, prior coronary artery bypass graft, PAD, current smoking, and renal dysfunction (estimated glomerular filtration rate <60 ml/min/1.73 m^2^). There was a significant risk gradient both in the full cohort and in the subgroup of patients with no prior heart failure (both p<0.001). TRS 2°P = TIMI Risk Score for Secondary Prevention.

Supplemental Figure S4**. Effect of Vorapaxar on Incidence of Hospitalization for Heart Failure by Major Clinical Subgroups.**

Cumulative incidence rates are expressed using 3-year Kaplan-Meier estimates. The FDA cohort included patients with qualifying diagnoses of PAD or prior MI and excluded those with prior stroke or transient ischemic attack. In this analysis, the TRS 2°P cohort was restricted to patients within the FDA cohort with a qualifying diagnosis of prior MI. Patients with MI were stratified based on whether the MI occurred <3 months, 3-6 months, or >6 months prior to enrollment. CI = confidence interval; HF = heart failure; IS = ischemic stroke; MI = myocardial infarction; mo = month; no. = number; PAD = peripheral artery disease; TRS 2°P = TIMI Risk Score for Secondary Prevention; yr = year.
